# Volvulus of the Sigmoid Colon Associated With Rectal Cancer: A Case Report

**DOI:** 10.14740/gr619w

**Published:** 2015-02-14

**Authors:** Seung-Hyun Lee, Byung-Kwon Ahn, Sung-Uhn Baek

**Affiliations:** a Department of Surgery Kosin University College of Medicine, Busan, Korea

**Keywords:** Intestinal volvulus, Sigmoid disease, Neoplasms, Rectum

## Abstract

Sigmoid volvulus is one of the three most common causes of acute colonic obstruction. Predisposing factors include chronic constipation, adhesion from a prior abdominal surgery, and megacolon. However, concomitant presentation of volvulus of the sigmoid colon and rectal cancer is extremely rare. We report a case of a 50-year-old woman with coexisting volvulus of the sigmoid colon and rectal cancer. The patient presented with abdominal distension and pain for 2 days. On computed tomography, the whole colon was dilated with gas and feces. A whirl sign with rotation of the inferior mesenteric vessel was identified. The rectum had irregular wall thickening. Colonoscopy showed a circumscribed, ulcerofungating mass approximately 6 cm from the anal verge. The sigmoid colon was obstructed at a point approximately 25 cm from the anal verge. The mucosa was hyperemic and edematous with the pathognomonic spiral pattern. Endoscopic reduction was not successful. On laparotomy, the sigmoid colon was rotated around its mesentery. It was severely distended with edematous, hyperemic serosa. A tumor of the rectum was identified in the mid-rectum. The patient underwent low anterior resection and protective ileostomy. Pathologic findings confirmed adenocarcinoma of the rectum. The postoperative course was complicated by an ileus, which was managed with conservative treatment.

## Introduction

Sigmoid volvulus is one of the three most common causes of acute colonic obstruction [1]. It refers to the twisting or torsion of the sigmoid colon about its mesentery. The etiology of this disorder is not completely understood. It is known to occur in the setting of a redundant sigmoid loop, which rotates around its narrow and elongated mesentery [2]. Predisposing factors include chronic constipation, adhesion from a prior abdominal surgery, and megacolon. However, presentation as coexisting sigmoid volvulus and rectal cancer is extremely rare. We report a case of concomitant volvulus of the sigmoid colon and rectal cancer.

## Case Report

A 50-year-old woman presented to the emergency department with lower abdominal pain and distension. Several months prior to admission, she experienced a change in her bowel habits with intermittent constipation and diarrhea. For 2 months, she also experienced intermittent bloody stool with a change in her stool caliber. Approximately 4 - 5 h prior to presentation, she experienced acute, severe lower abdominal pain. She did not complain of nausea and vomiting. She had a past surgical history of a cesarean section approximately 20 years ago.

On admission, the patient’s height, weight, and body mass index were 145 cm, 40 kg, and 19.0, respectively. She had a weight loss of approximately 10 kg over the past year. Vital signs were normal with a blood pressure of 110/80 mm Hg, pulse rate of 52 beats/min, respiration rate of 20/min, and body temperature of 36.7 °C. Physical examination revealed tenderness and rigidity in the left lower abdominal quadrant. Intestinal sounds were strongly audible with increased frequency on auscultation. Laboratory investigation revealed the following: white blood cell count, 7,510/mm^3^ (63.9% neutrophils); hemoglobin, 9.9 g/dL; hematocrit, 32.1%; platelet count, 323 × 10^3^/mm^3^; serum protein, 6.5 g/dL; serum albumin 3.7 g/dL; total bilirubin, 0.36 mg/dL; alanine aminotransferase, 11 IU/L; aspartate aminotransferase, 7 IU/L; alkaline phosphatase, 79 U/L; amylase, 46 U/L; lipase, 19 U/L; creatinine, 0.58 mg/dL; serum Na, 134 mEq/L; serum K, 3.2 mEq/L; and carcinoembryonic antigen, 1.70 ng/mL.

**Figure 1:**
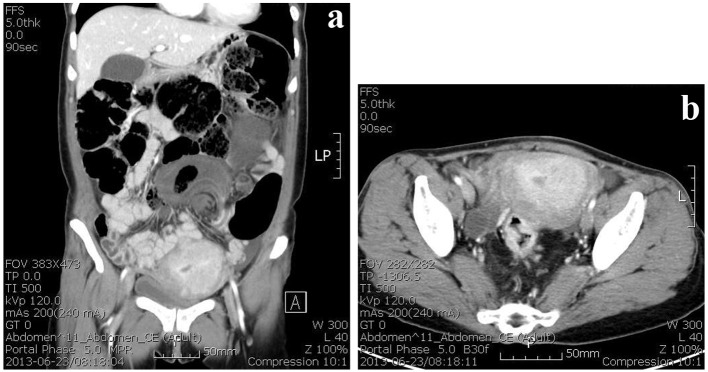
CT findings show whirl sign with rotation of inferior mesenteric vessel was identified (a). The rectum had irregular wall thickening (b).

On computed tomography, the whole colon was dilated with gas and feces. A whirl sign with rotation of the inferior mesenteric vessel was identified (Fig. 1a). The rectum had irregular wall thickening (Fig. 1b). Colonoscopy showed a circumscribed, ulcerofungating mass approximately 6 cm from the anal verge. The sigmoid colon was obstructed at a point approximately 25 cm from the anal verge. The mucosa was hyperemic and edematous with the pathognomonic spiral pattern of a volvulus. Endoscopic reduction was not successful.

**Figure 2:**
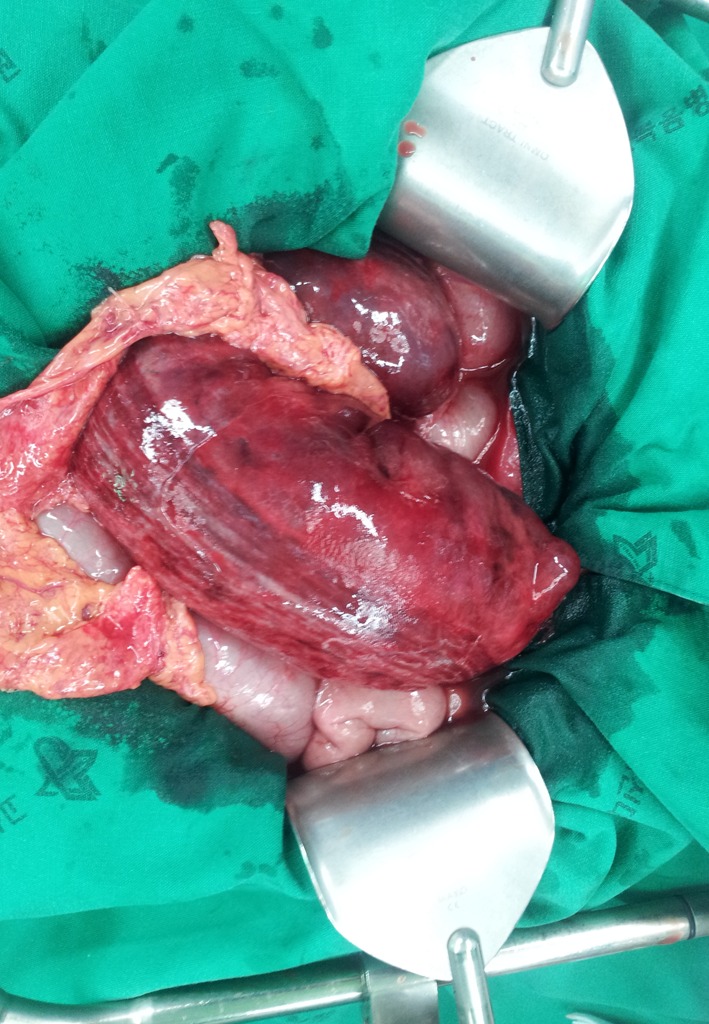
Operative finding shows the sigmoid colon rotation around its mesentery about 270°. It has severely dilatation with edematous, hyperemic serosa.

On laparotomy, the sigmoid colon was rotated around its mesentery approximately 270°. It showed severe dilatation with edematous, hyperemic serosa (Fig. 2). A tumor of the rectum was identified in the mid-rectum. The remainder of the large bowel was unremarkable. The patient underwent low anterior resection and protective ileostomy. Pathologic findings confirmed adenocarcinoma of the rectum. On pathologic examination, the tumor invaded to the pericolic fat tissue. The tumor was identified in four lymph nodes out of a total of 21 sampled lymph nodes. The postoperative course was complicated by an ileus, which was managed with conservative treatment.

## Discussion

Sigmoid volvulus refers to the twisting or torsion of the sigmoid colon about its mesentery. It comprises 50-90% of all cases of colonic volvulus [1]. The etiology of this disorder is not completely understood. A long redundant sigmoid colon with a narrow mesentery is a predisposing condition for sigmoid volvulus formation. Chronic fecal overloading is believed to cause elongation and dilatation of the sigmoid colon [2]. Chronic fecal overloading is related to chronic constipation and adhesion from a prior abdominal surgery. A coexistent colon tumor may be considered as a predisposing factor for colon volvulus, which is associated with aggravating proximal bowel dilatation. In this report, the patient had two predisposing factors, including a medical history of previous abdominal surgery and a concurrent colon tumor. Operative findings showed no significant intra-abdominal adhesions, which can be a cause of chronic fecal overloading.

The presentation of colon volvulus with a coexistent tumor of the colon is extremely rare. Lapin et al [3] reported a case of a 36-year-old woman with a volvulus of the transverse colon associated with a submucosal hamartoma. The patient had chronic constipation with a family history of Von Recklinghausen’s disease. The submucosal hamartoma was found in a resection specimen of the transverse colon. Meyers et al [4] reported a case of a 54-year-old man with a cecal volvulus and an obstructive carcinoma of the left colon. Figiel and Figiel [5] reported a case of an 80-year-old woman with ascending colon volvulus and a coexistent carcinoma at the splenic flexure. Wecksell and Gordon [6] reported a case of an 80-year-old man with an ascending colon volvulus associated with an adenocarcinoma of the transverse colon. Natarajan et al [7] reported a case of a 70-year-old woman with a cecal volvulus and a coexistent carcinoma at the rectosigmoid colon. The last three cases were cecal or ascending colon volvulus with an age range of 70 - 80 years old. This is the first case report of a sigmoid volvulus with concurrent rectal tumor.

Based on our case review, it is important for the surgeon to examine the entire colon when operating on cases of colon volvulus to identify concurrent colon tumors.
